# Optimization and characterization studies of poultry waste valorization for peptone production using a newly Egyptian *Bacillus subtilis* strain

**DOI:** 10.1186/s13568-024-01794-1

**Published:** 2025-01-13

**Authors:** Hajar Saeed, Anthony Ragaey, Ziad Samy, Viola Ashraf, Aly ElMostafa, Norhan Ahmad, Enjy Bebawy, Nour ElHoda M. Sorour, Salwa M. El-Sayed, Ashraf Bakry, Naglaa Ebeed, Hesham Elhariry, Thanaa El-Noby, Samah H. Abu-Hussien

**Affiliations:** 1 Biotechnology Program, New Programs Administration, Faculty of Agriculture, Ain Shams University, Hadayek Shoubra, P.O. Box 68, Cairo, 11241 Egypt; 2 Department of Genetics, Faculty of Agriculture, Ain Shams University, Hadayek Shoubra, P.O. Box 68, Cairo, 11241 Egypt; 3 Department of Agricultural Biochemistry, Faculty of Agriculture, Ain Shams University, -Hadayek Shoubra, P.O. Box 68, Cairo, 11241 Egypt; 4 Department of Food Science, Faculty of Agriculture, Ain Shams University, Hadayek Shoubra, P.O. Box 68, Cairo, 11241 Egypt; 5 Department of Agricultural Economics, Faculty of Agriculture, Ain Shams University, Hadayek Shoubra, P.O. Box 68, Cairo, 11241 Egypt; 6 Department of Agricultural Microbiology, Faculty of Agriculture, Ain Shams University, Hadayek Shoubra, P.O. Box 68, Cairo, 11241 Egypt

**Keywords:** Poultry waste valorization, Peptone production, *Bacillus subtilis*, Amino acids, Proteases

## Abstract

Valorization of poultry waste is a significant challenge addressed in this study, which aimed to produce cost-effective and sustainable peptones from poultry waste. The isolation process yielded the highly potent proteolytic *B.subtilis* isolate P6, identified through 16S rRNA gene sequencing to share 94% similarity with the *B.subtilis* strain KEMET024 (GenBank accession number PP694485.1) and deposited in MIRCEN culture collection, Cairo, Egypt as EMCC 998871. It reached optimal production levels during 24 h of incubation, with biomass at 2.5 g/L, protease activity at 455 U/mL, and total amino acid (TAA) concentration at 208 mg/mL. For screening the most significant factors for peptone production, the Plackett–Burman design identified meat and bone meal concentration as the main significant factor influencing total amino acid reaching 420 mg/mL. BOX-Behnken design optimized peptone production increasing its production level by twofold to reach 2850 U/mL of protease activity and 580 mg/mL of total amino acids. The produced peptone demonstrated a superior amino acid profile compared to commercial peptones, with a remarkably higher total amino acid content of 621.556 mg/g and elevated levels of essential amino acids like aspartic acid (37.745%), glutamic acid (90.876%), glycine (117.272%), and alanine (50.373%). Characterization revealed optimal pH and temperature conditions of around pH 8 and 50–60°C, respectively, for the proteolytic activity. The Michaelis–Menten and Lineweaver–Burk plots determined a Km of 0.5 mg/mL and Vmax of 174.08 U/mL suggesting cooperative substrate binding and providing insights into the enzyme’s maximum rate and affinity. The produced peptone exhibited minimal cytotoxicity at lower concentrations (≤ 1 mg/mL), with cell viability exceeding 94% against normal human skin fibroblast (HSF) cells. However, higher concentrations (≥ 3 mg/mL) displayed increased cytotoxic effects. Moreover, the results strongly indicate that the produced peptone, particularly at 0.5% concentration, is an effective nitrogen source for *B. subtilis* cultivation, demonstrating its potential for biotechnological applications. This study successfully valorized poultry waste by developing a sustainable and cost-effective alternative to commercial peptones, contributing to waste valorization and sustainable biotechnological processes.

**Graphical abstract:**

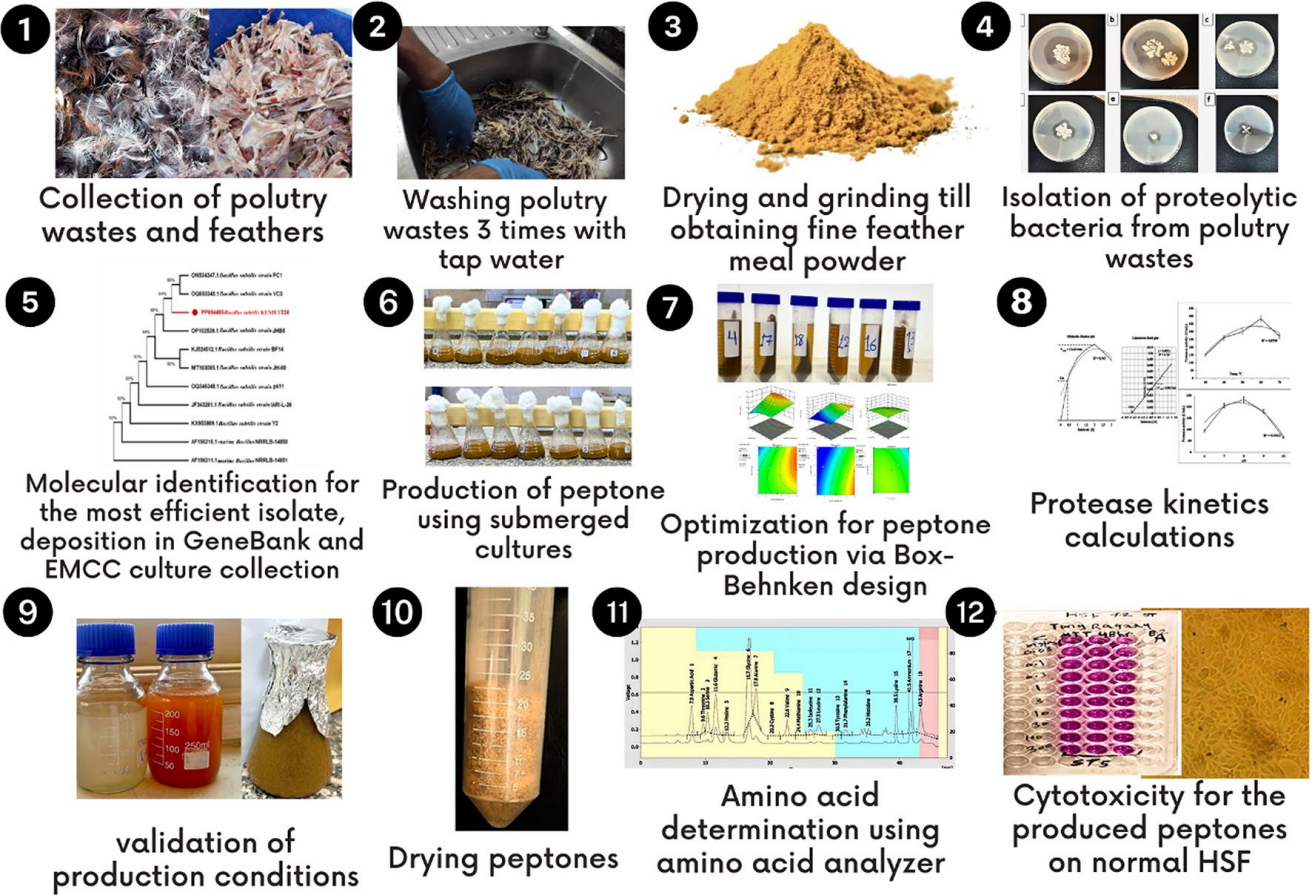

**Supplementary Information:**

The online version contains supplementary material available at 10.1186/s13568-024-01794-1.

## Introduction

The poultry industry generates enormous quantities of waste materials, posing a significant environmental challenge and economic burden (Karuppannan 2020). According to estimates, global poultry production results in over 24 million tons of chicken feather waste annually (Tesfaye 2017). This feather waste represents a rich source of proteins, particularly keratin, a fibrous structural protein that constitutes over 90% of the feather's composition (Chukwunonso Ossai 2022). However, the recalcitrant nature of keratin, due to its tightly packed and cross-linked structure, makes it remarkably resistant to degradation by common proteolytic enzymes (El-Ghonemy and Ali 2021). As a result, the accumulation of this proteinaceous waste poses significant environmental and health risks if not managed properly (Peydayesh 2023).Traditionally, poultry feather waste has been disposed of through landfilling, incineration, or low-value applications in animal feed or fertilizers (McGauran 2021). However, these methods are often unsustainable, and inefficient, and may contribute to environmental pollution, such as greenhouse gas emissions, water contamination, and soil degradation.The high protein content of feather waste, which could be a valuable resource, remains underutilized in these conventional disposal methods.In recent years, there has been growing interest in exploring sustainable and eco-friendly approaches to valorize feather waste through biotechnological processes (Falade 2021). One promising avenue is the microbial conversion of feather waste into value-added products, such as enzymes, animal feed additives, and biofertilizers, the production of peptones, complex mixtures of peptides, amino acids, and nitrogenous compounds, has gained significant attention due to their widespread applications in various industries, including biotechnology, pharmaceuticals, and food (Chaturvedi 2021). Peptones are essential components in microbiological media, serving as sources of nitrogen, vitamins, and growth factors for the cultivation of a wide range of microorganisms, they are also used as supplements in animal feed, cosmetics, and other industrial processes (Veerapandian 2020). Traditionally, peptones have been derived from animal-based protein sources, such as meat, milk, and casein, through enzymatic or acid hydrolysis, and the increasing demand for peptones, coupled with the rising costs and sustainability concerns associated with conventional sources, has prompted the exploration of alternative and renewable feedstocks (Lezin 2022).The microbial production of peptones from feather waste offers a sustainable and economically viable solution to address the challenges of waste management and the increasing demand for peptones. This approach leverages the ability of certain microorganisms, particularly proteolytic bacteria and fungi, to degrade and hydrolyze the recalcitrant keratin present in feather waste, thereby releasing amino acids and peptides (Karuppannan 2020; Sahoo 2023). The utilization of these microorganisms in fermentation processes not only facilitates the valorization of feather waste but also contributes to the development of sustainable and cost-effective methods for peptone production (Srivastava 2020). However, the microbial production of peptones from feather waste is a complex process that involves several key steps, including the isolation and screening of proteolytic microorganisms, optimization of fermentation conditions, and downstream processing for peptone extraction and purification (Stiborova 2016). Each of these steps presents its challenges and requires careful investigation and optimization to ensure efficient and cost-effective peptone production (Imron 2020).Despite the potential benefits and the ongoing research efforts, several gaps and challenges remain in the development of a scalable and commercially viable process for the microbial production of peptones from feather waste, one significant challenge is the identification and selection of highly potent proteolytic microorganisms capable of efficiently degrading the recalcitrant keratin structure present in feather waste (Nayak 2024). While various microorganisms have been explored for their keratinolytic and proteolytic activities, the search for more efficient and robust strains with enhanced enzymatic capabilities is an ongoing endeavour (Hassan 2020).Another critical challenge lies in the optimization of fermentation conditions, including medium composition, pH, temperature, and aeration, which play crucial roles in maximizing peptone yield and quality the complex nature of feather waste and the intricate interactions between various fermentation parameters necessitate the use of advanced statistical techniques and experimental design approaches to identify the most significant factors and determine their optimal combinations (Kasemiire 2021). Furthermore, the downstream processing steps, such as separation, purification, and concentration, are essential for obtaining high-quality peptones suitable for various applications (John 2020). Developing efficient and cost-effective strategies for recovering and purifying the desired peptone fractions from the fermentation broth remains a significant challenge, particularly when considering the potential for scaling up the process to industrial levels (Dey).In addition to the technical challenges, the potential applications and commercial viability of the produced peptones need to be thoroughly evaluated and characterized( O’Neill 2021).This includes assessing the amino acid composition, molecular weight distribution, functional properties, and potential toxicological effects of the peptones, as well as exploring their suitability for various applications, such as microbial growth media, food additives, and pharmaceutical applications( Lassoued 2015). The microbial production of peptones from poultry feather waste presents a promising and sustainable approach to address the challenges of waste management and the increasing demand for peptones and some significant gaps and challenges need to be addressed to develop a scalable and commercially viable process( Sypka 2021). This includes isolating highly potent proteolytic microorganisms, optimizing fermentation conditions, developing efficient downstream processing strategies, and thoroughly characterizing the produced peptones for potential applications( Kamal 2017). Overcoming these challenges requires a multidisciplinary approach involving microbiology, biotechnology, bioprocess engineering, and analytical techniques( Neethirajan 2018). By addressing these gaps, this research aims to isolate and identify a highly potent proteolytic bacterial strain capable of efficiently hydrolyzing poultry feather waste. Subsequently, it seeks to optimize the fermentation conditions for the microbial production of peptones from feather waste using the selected bacterial strain. Additionally, the study evaluates the potential applications and toxicological effects of the produced peptones, particularly their suitability as a microbial growth medium and their cytotoxicity against human cell lines.

## Materials and methods

### Sample collection

Twenty-five samples of poultry waste (feathers, meat, and bones) were collected in sterile plastic bags from slaughterhouses in Cairo, Egypt. The samples were transported under cooling to the New Programs Administration, Biology lab, Faculty of Agriculture, Ain Shams University, Cairo, Egypt on ice and stored at 4°C for further studies.

### Microorganisms and media used

*B. subtilis* ATCC 6051 (https://www.atcc.org/products/6051) was used as a strain for skim milk hydrolysis. It was obtained from the Microbial Resources Center (MIRCEN) located in Cairo, Egypt. Tryptic Soy Broth (TSB) was used for cultivating all obtained isolates. It has the following composition (Pancreatic digest of casein: 17.0 g/L, Papaic digest of soybean meal: 3.0 g/L, Sodium chloride: 5.0 g/L, Dipotassium phosphate: 2.5 g/L, Glucose: 2.5 g/L) (Joardar and Rahman 2018). Skim Milk Agar, was used for the detection and enumeration of proteolytic bacteria. It has the following composition(Skim milk powder: 28.0 g/L, Tryptone: 5.0 g/L, Glucose: 1.0 g/L, Yeast extract: 2.5 g/L, Agar 15.0 g/L) Skim Milk Agar (DM613). All chemicals were of fine grade.

### Isolation of proteolytic *bacteria*

For isolation of proteolytic bacteria, 100 µL of each of all waste samples were spread plated onto skim milk agar plates containing 2% skim milk powder, 0.5% peptone, and 1.5% agar. The inoculated plates were incubated under aerobic conditions at 30℃ for 48 h. After incubation, the skim milk agar plates were inspected for zones of clearance. Hydrolysis zones were measured in cm and the most efficient isolate was selected and transferred onto new skim milk agar plates through streak plating to obtain pure culture isolates (Nassar 2015a). The selected isolate P6 was maintained on nutrient agar slants at 4℃ and subcultured at monthly intervals and for further studies.

### Standard inoculum

For standard inoculum preparation, the selected P6 isolate was streaked and grown on TSB at 30℃ for 24h. 50 mL of TSB were inoculated with a single colony of P6 isolate and incubated at 30℃ for 24 h at an agitation speed of 150 rpm yielding a final working inoculum of approximately 1 × 10^6^ CFU/mL (Abu-Hussien and Mohamed 2020).

### Phenotypic identification of the selected P6 isolate

The identification of the bacterial isolates was done by examining their phenotypic characteristics. This involved observing the properties of the bacterial colonies on culture media as well as studying the morphological features of the individual bacterial cells under the microscope. To facilitate the examination of cell morphology, the bacterial isolates were subjected to Gram staining and spore staining techniques. ( Pradhan and Tamang 2019).

### Molecular identification of the P6 isolate

The bacterial DNA was extracted, and a molecular approach utilizing the polymerase chain reaction (PCR) technique was employed for gene sequencing. The 16S rRNA gene sequence was partially amplified using two universal primers of 27F (5′AGAGTTTGATCCTGGCTCAG3′) and 1492R (5′TACG GCTACCTTGTTACGACTT3′). The partially amplified PCR product was purified using a QIA quick gel extraction kit (Qiagen, Germany). The purified PCR product containing the 16S rRNA sequence was sent to Macrogen company (South Korea) for sequencing. The sequence readings were clipped and assembled using BioEdit version 7.0.4. ClusterW version 4.5.1 was utilized to align the resulting genomic information. BLAST inquiries were carried out against the NCBI database to identify the bacterial isolates. Phylogenetic trees were constructed using the neighbor-joining method with the MEGA 11 software. The MEGA software (version 11.0) is available from https://www.megasoftware.net/(Abd-Elhalim 2023b).

### Time course of peptone production

The prepared standard inoculum of *B. subtilis* strain KEMET024 isolate was inoculated into TSB medium at a concentration of 5% v/v (2 × 10^7 CFU/mL) and incubated at 30°C for 48 h with shaking at 150 rpm. At intervals of 6h, 10 mL samples were collected from the cultures. These samples were centrifuged at 10,000 rpm for 15 min. The resulting pellets were used for determining the cell dry weight (CDW), while the supernatants were collected to evaluate Protease activity (U/mL), and total amino acids (mg/mL). All experiments were performed in triplicate (Nassar 2015a).

### Optimization for peptone production using the selected *B.subtilis* strain KEMET024

#### Statistical screening using the Plackett–Burman design

A Plackett–Burman experimental design as shown in Tables (S1, and S2) was carried out to screen 11 nutritional and environmental factors (A: Feather meal, B: Meat and bone meal, C: Starch, D: Casein, E: CaCO_3_, F: KH_2_PO_4_, G: pH, H: Temperature, J: Inoculum size, K: Agitation, and L: Incubation time) for their effects on the peptone production by the selected isolate. (Abu-Hussien and Mohamed 2020; Thiruchelvi 2020). Independent variables were studied at high (+ 1) and low (-1) levels along with center points (0) in a two-level fractional factorial design consisting of 12 runs and 5 additional center points. All trials were carried out in triplicates. Significant factors from the Plackett–Burman design were identified based on the magnitude and direction of their main effect coefficient and corresponding p-values. For peptone extraction and amino acids determination, the fermented medium was centrifuged at 6000 rpm for 15 min at 4ºC to obtain amino acids-rich broth., the supernatant was stored at 4ºC for analysis and the assay was done within 24 h. as described below. For Protease activity and protein content, the protein content was determined by the method of as described later. (Nassar 2015b).

### Box-Behnken design for peptone optimization using P6 isolate

A three-factor, three-level Box-Behnken design as shown in Table (S3) was implemented to optimize peptone production by assessing the effects and interactions of meat and bone waste (5, 7.5, and 10 g/L), starch (0, 2.5, and 5 g/L), and CaCO_3_ (0, 0.25, and 0.5 g/L) through response surface methodology quadratic model fitting (Nassar 2015c). The complete design consisted of 17 experimental runs with the independent variables set at low, middle, and high values. All trials were carried out in triplicates. Statistical analysis of the experimental data enabled the modeling of peptone yield to determine the optimum formulation through the evaluation of the quadratic effects and interactions between the studied nutrient components.

### Cytotoxicity of produced peptone against normal human skin fibroblast

Human skin fibroblast (HSF) cells acquired from Nawah Scientific Inc. (https://nawah-scientific.com) were cultivated in Dulbecco's Modified Eagle's Medium (DMEM) supplemented with 10% fetal bovine serum, 100 mg/mL streptomycin and 100 U/mL penicillin at 37°C in a 5% CO_2_ humidified atmosphere (Abd-Elhalim 2023a). The sulforhodamine B (SRB) assay was utilized to evaluate the cytotoxicity of metabolites obtained from P6 isolate by quantifying HSF cell viability after treatments (Mansour 2023).

### Protease activity

The activity of proteases was evaluated using a modified version of (Anson 1938) method. A 1 mL sample of the crude enzyme solution was mixed with 5 ml mL of casein solution and incubated for 10 min at 37°C. After incubation, the reaction was terminated by adding 5 ml mL of 0.11M trichloroacetic acid (TCA) solution. Following 30 min, the mixture was centrifuged at 10,000 rpm for 15 min. Two mL of the supernatant was then combined with 5 mL of 0.5M sodium carbonate and 1 mL of Folin-Ciocalteu's Phenol reagent and allowed to stand for 30 min. at room temperature. All trials were carried out in triplicates. The optical densities of the solutions were measured against a blank at 660 nm. The blank sample consisted of the same components, except that the enzyme solution was substituted with distilled water. The readings were completed within 30 min. The casein solution (0.65% w/v) was prepared by dissolving 6.5 mg/mL of casein in 50 mM potassium phosphate buffer. The solution was gradually heated to 80–85°C for about 10 min, with gentle stirring, to achieve a homogeneous dispersion. The pH was adjusted, if necessary, with NaOH and HCl. A tyrosine standard solution in the range of 0–1000 mg/L was prepared in triplicate to obtain a standard curve. One unit (U) of protease activity was defined as the amount of enzyme that produced 1μg of tyrosine in 10 min. under the specified assay conditions.( Nassar et al. 2015b).

### Total amino acids

The crude amino acids were first separated from the bacterial culture by centrifugation at 10,000 rpm under cooled conditions for 15 min. The cell pellets were collected to measure the dry cell weight, while the supernatant containing the extracellular amino acids was used for amino acid identification and analysis. The quantification of the total amino acid content was performed using the ninhydrin method outlined by (Anson 1938). All trials were carried out in triplicates. This colorimetric assay involves mixing 1 mL of the sample with 1 mL of ninhydrin reagent solution, which is prepared by dissolving 2–4% (w/v) ninhydrin powder in a sodium acetate buffer and ethanol. A standard solution of glycine is prepared at various concentrations (e.g., 0.1 to 1.0 mM) to create a calibration curve. After mixing the sample and ninhydrin reagent, the reaction is carried out by heating at 100°C for 30 min. to allow color development, where ninhydrin reacts with amino groups to form a purple-colored complex (Ruhemann's purple). The colored solution is measured at 570 nm and compared to the standard curve to determine the total amino acid concentration in the sample.

### Amino acid profile using amino acid analyzer

The analysis of amino acids was performed using a Sykam Amino Acid Analyzer (Sykam GmbH, Germany). The analyzer was equipped with a Solvent Delivery System S 2100 (Quaternary pump with a flow range of 0.01 to 10.00 mL/min and a maximum pressure of up to 400 bar), Autosampler S 5200, Amino Acid Reaction Module S4300 (with a built-in dual filter photometer between 440 and 570 nm, providing constant signal output and signal summary option), and Refrigerated Reagent Organizer S 4130. For standard sample preparation, A stock solution containing 18 amino acids (aspartic acid, threonine, serine, glutamic acid, proline, glycine, alanine, cystine, valine, methionine, isoleucine, leucine, tyrosine, phenylalanine, histidine, lysine, ammonia, and arginine) was prepared. The concentration of all amino acids, except cystine, was 2.5 μMol/mL, while the concentration of cystine was 1.25 μMol/mL. For analysis, 60 μL of the stock solution was diluted in a 1.5 mL vial with a sample dilution buffer. The diluted solution was then filtered using a 0.45 μm syringe filter, and 100 μL of the filtered solution was injected into the analyzer. For sample preparation, 0.531 g of the sample was transferred into a seal and digested with 25 ml of 6 N HCl at 100°C for 24 h. After cooling, the solvent was evaporated using a Rota Vab. The samples were then dissolved in 10 mL of dilution buffer and sonicated for 15 min. The volume was made up to 50 mL in a volumetric flask. Subsequently, 1 mL of this solution was diluted with 20 mL of dilution buffer, filtered using a 0.45 μm PTFE syringe filter, and 100 μL of the filtered solution was injected into the analyzer. The following parameters were set for the amino acid analysis: LCA K06/Na column, mobile phases comprising Buffer A, Buffer B, and Regeneration solution, gradient elution mode, a flow rate of 0.45 mL/min, a temperature gradient from 57°C to 74°C, and detection wavelengths of 440 nm and 570 nm (Abu-Hussien and Mohamed 2020).

### Effect of poultry waste substrate concentration on enzyme velocity

To determine the kinetic parameters Vmax and Km, the effect of substrate concentration on protease activity was investigated. The crude enzyme was incubated with varying concentrations of poultry waste (0, 0.5, 1, 1.5, 2, 2.5, 3g/L) in the reaction mixture and assayed under standard conditions at each concentration. Enzyme activity per unit of time was measured at each substrate level. All trials were carried out in triplicates. The data obtained was plotted using both the Michaelis–Menten and Lineweaver–Burk models to calculate the values of Km and Vmax. By assaying enzyme activity across a range of substrate concentrations and graphing the data, the maximum velocity (Vmax) and Michaelis constant (Km) kinetics values for the protease enzyme could be determined.(Mousami Shankar et al. 2018).

### Application of produced peptone from *B. subtilis* strain KEMET024 as the sole source of nitrogen

The ability of the produced peptone to serve as a sole nitrogen source was evaluated by cultivating *B.subtilis* ATCC 6051 in a minimal medium supplemented with varying concentrations of peptone. The minimal medium was prepared with the following composition (per liter): 6.8 g Na_2_HPO_4_, 3.0 g KH_2_PO_4_, 0.5 g NaCl, 1.0 g NH_4_Cl, 0.24 g MgSO_4_, 0.01 g CaCl_2_, and 4.0 g glucose. For the nitrogen source evaluation, NH_4_Cl was omitted from the minimal medium, and the produced peptone was added at concentrations of 0.1%, 0.3%, 0.5%, and 1.0% (w/v). A control culture without any supplemented nitrogen source was also included. Each culture was inoculated with a single colony of *B.subtilis* from a fresh TSA plate and incubated at 30°C for 24h with shaking at 150 rpm. The growth of the cultures was monitored by measuring the cell dry weight at regular intervals over 24h. The ability of the peptone to support bacterial growth was evaluated by comparing the growth curves of the cultures with different peptone concentrations to the control culture without any supplemented nitrogen source(Gray et al. 2008). All trials were carried out in triplicates.

### Statistical analysis

Data were analyzed by one-way ANOVA followed by Tukey's post-hoc test using SPSS 12. *P* < *0.05* was considered statistically significant. A Tukey test at a P-value of 0.05 was applied.( Keselman and Rogan 1977) All samples and collected data were statistically analyzed using Design Expert 12 Statistics software (https://www.statease.com/software/design-expert).

## Result

### Isolation of proteolytic *bacteria*

As shown in Table 1, The isolates P3, P6, P4, and P5, which were identified as long rods, chained, Gram-positive, and spore-formers, exhibited higher protease activity on both skim milk agar and feather agar compared to the control strain *B. subtilis* ATCC 6051. Notably, isolates P3 and P6 showed significantly higher zones of hydrolysis on skim milk agar (2.63 ± 0.15 cm and 2.46 ± 0.35 cm, respectively) than the control (1.6 ± 0.15 cm). On feather agar, isolates P4, P5, and P6 displayed remarkably higher protease activity, ranging from 8.00 ± 0.7 to 8.25 ± 0.35 cm, while the control strain showed no activity. These findings suggest that the isolated strains, particularly P3, P4, P5, and P6, have greater potential for protease production compared to the control strain *B. subtilis* ATCC 6051.

**Table 1:** Proteolytic activity of bacterial isolates from poultry waste on skim milk and feather agar

Hydrolysis zone (cm)			Morphological characters	
Isolate No	Skim milk agar	Feather agar		
P1	0.57 ± 0.35	0.00 ± 0.00	Long rods, chained, Gram +, spore former	
P2	0.00 ± 0.00	0.00 ± 0.00	Cocci, Gram +, non spore former	
P3	2.63 ± 0.15	7.75 ± 0.35	Long rods, chained, Gram +, spore former	
P4	1.93 ± 0.11	8.25 ± 0.35	Long rods, chained, Gram +, spore former	
P5	1.70 ± 0.10	8.00 ± 0.7	Long rods, chained, Gram +, spore former	
P6	2.46 ± 0.35	8.00 ± 0.7	Long rods, chained, Gram +, spore former	
P7	0.00 ± 0.00	0.00 ± 0.00	Cocci, Gram +, non spore former	
P8	0.00 ± 0.00	0.00 ± 0.00	Cocci, Gram +, non spore former	
P9	0.00 ± 0.00	0.00 ± 0.00	Cocci, Gram +, non spore former	
P10	0.00 ± 0.00	0.00 ± 0.00	Cocci, Gram +, non spore former	
*B. subtilis* ATCC 6051 (control)	1.6 ± 0.15	0.00 ± 0.00	Long rods, chained, Gram +, spore former	

For phenotypic identification, Table 1 includes morphological characteristics for each of the bacterial isolates tested. Several isolates (P1, P3, P4, P5, P6, and the *B. subtilis* ATCC 6051 control were identified as long rods arranged in chains, being Gram-positive, and forming endospores. In contrast, P2, P7, P8, P9, and P10 isolates were characterized as Gram-positive cocci that did not form endospores.

### Molecular identification of the P6 isolate

**Fig. 1:**
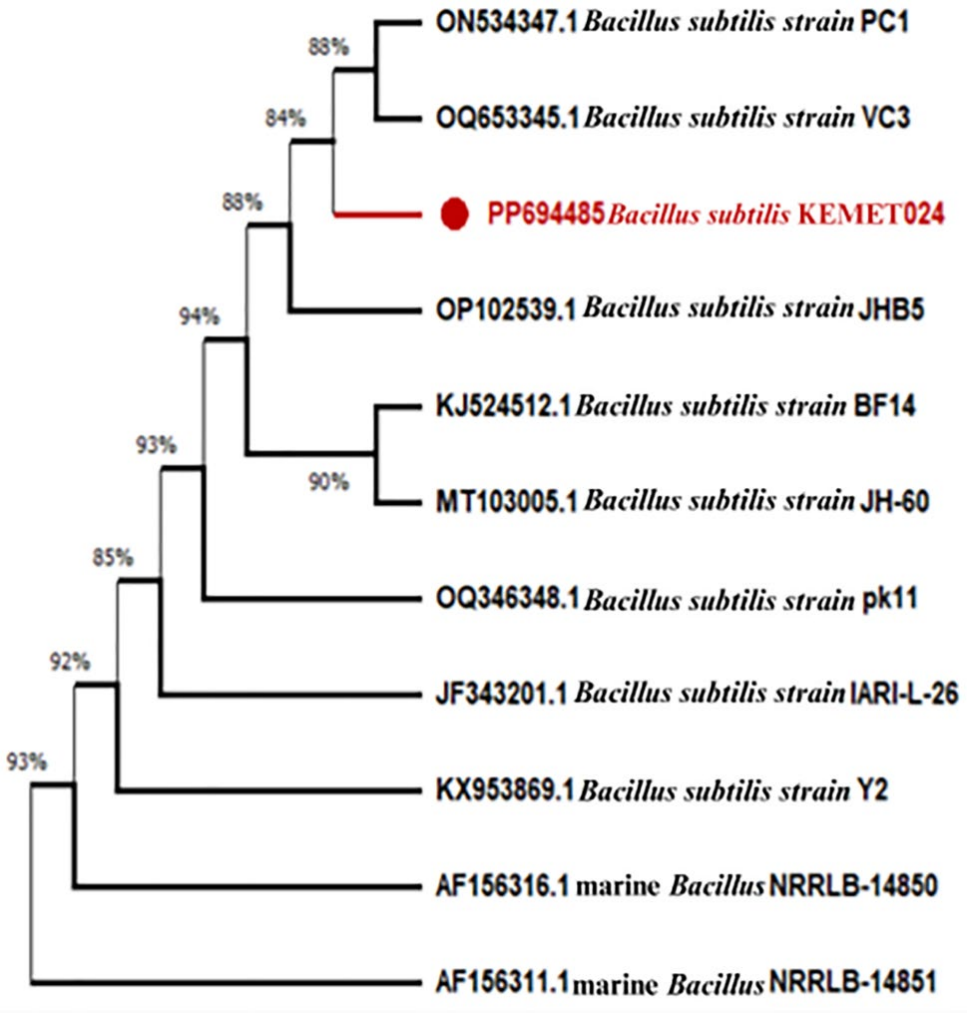
Phylogenetic analysis of *B. subtilis* KEMET024 (PP694485.1) and related *Bacillus* species based on 16S rRNA gene sequences

The bacterial DNA of the P6 isolate was extracted, and the 16S rRNA gene sequence was partially amplified through polymerase chain reaction (PCR). The purified PCR product containing the 16S rRNA was sequenced. The obtained sequence readings were clipped, assembled using BioEdit, and aligned with ClusterW. BLAST searches against the NCBI database were performed to identify the bacterial isolates. Phylogenetic analysis in Fig. 1 was conducted using the neighbor-joining method in MEGA 11 software (available from https://www.megasoftware.net/). The analysis revealed that the *Bacillus* P6 isolate was identified as a *B. subtilis* strain and deposited in GenBank as *B. subtilis* KEMET024, and under the accession number PP694485.1 (https://www.ncbi.nlm.nih.gov/nuccore/PP694485.1), shared a 94% similarity with OP102539. However, it was distinctly separated from the marine *Bacillus* strains AF156316.1, marine *Bacillus* NRRLB-14850, and AF156311.1 marine *Bacillus* NRRLB-14851, which formed a separate clade.

### Time course of peptone production

**Fig. 2:**
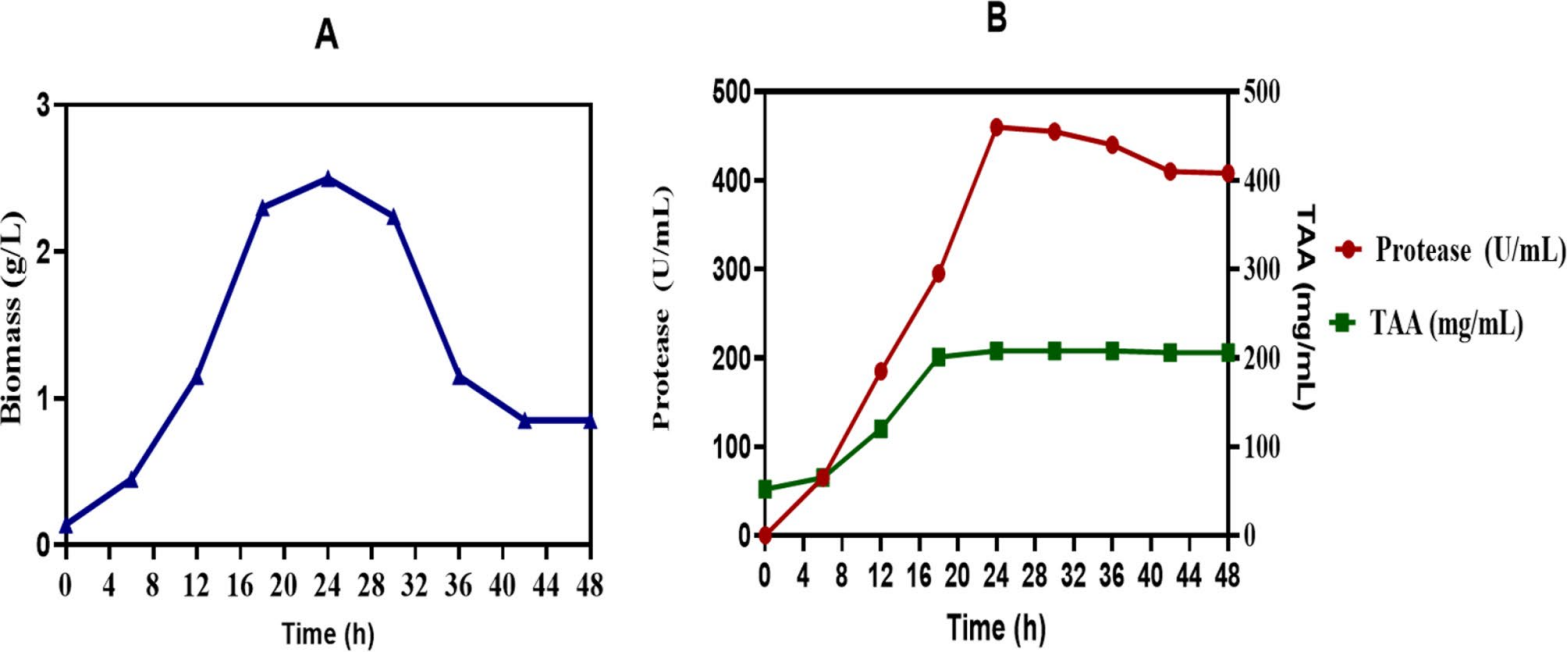
Effect of incubation time on biomass, total amino acids and protease production by *B. subtilis* KEMET024 incubated at 30 ℃ for 48h at 150 rpm

Figure 2 illustrates the effect of incubation time on biomass production (g/L), protease activity (U/mL), and total amino acid (TAA) (mg/mL) levels by *Bacillus subtilis* KEMET024 over a 48-h period at 30°C with shaking at 150 rpm. The biomass increased gradually, peaking at 2.5 g/L at 24 h. After this peak, biomass declined, stabilizing at 0.85 g/L by 48 h. Protease production displayed a sharp increase during the initial 24 h, reaching a maximum of approximately 455 U/mL, after which it declined in the latter stages of incubation. TAA production followed a similar trend, rising to 208 mg/mL by 24 h and then remaining relatively stable for the rest of the incubation period.

## Optimization for peptone production using *B. subtilis* Strain KEMET024

### Statistical screening using the Plackett–Burman design

**Fig. 3:**
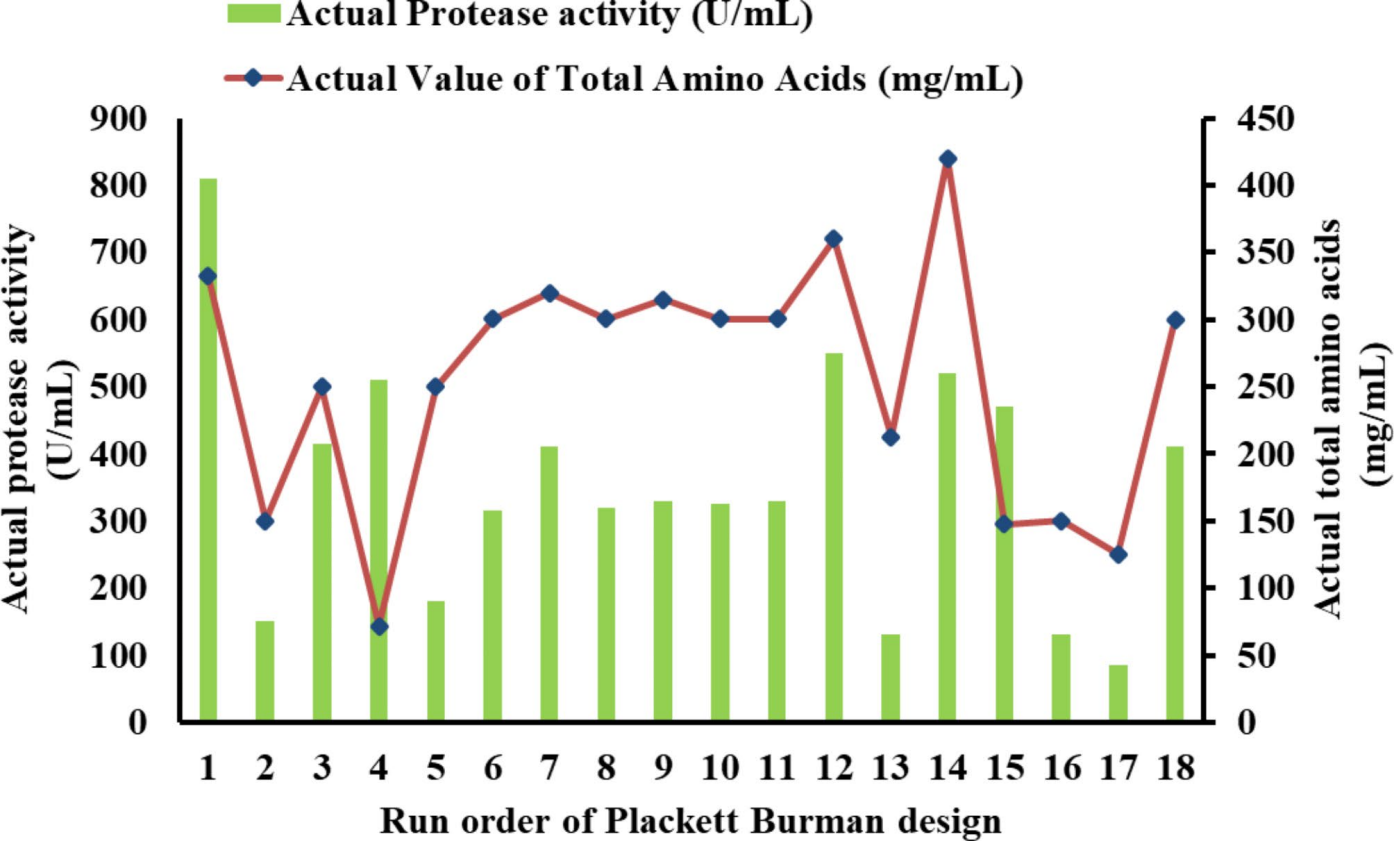
Actual protease activity, total amino acids, and biomass from across Plackett–Burman experimental runs

**Fig. 4:**
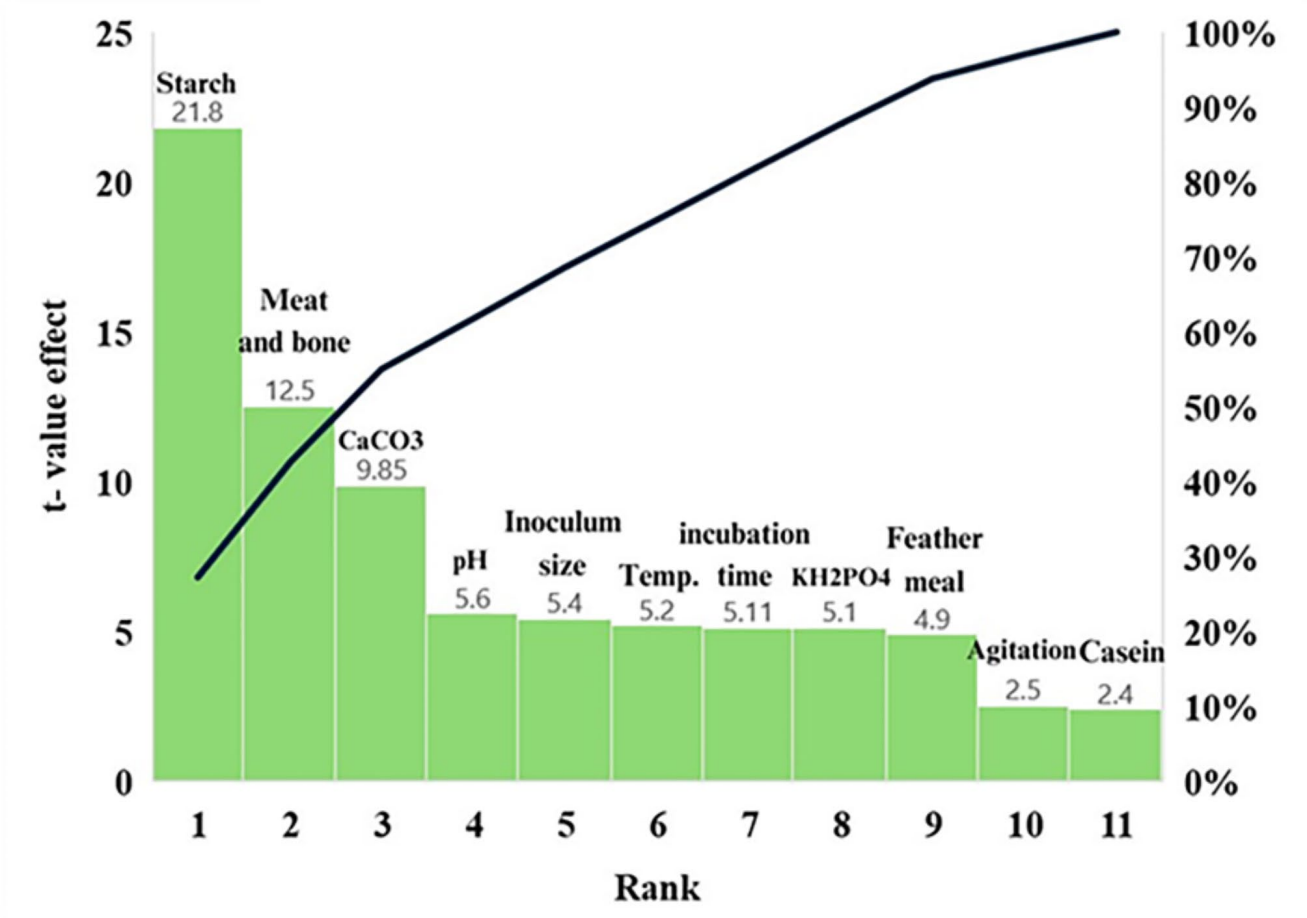
Pareto chart illustrating the relative effects of various factors on TAA and protease production from *B. subtilis* KEMET024 ranking meat and bone concentration as the most significant nitrogen factor

The results of the 18 experimental runs using the Plackett–Burman design depicted in Fig. 3 show a positive relationship between protease activity and biomass, suggesting that higher biomass may lead to increased protease production. However, (TAA) concentration follows a different pattern, peaking in specific run14 independent of protease and biomass trends reaching 420 mg/mL. The Pareto chart depicted in Fig. 4 confirms the model's statistical significance, with A-meat and bone identified as the most influential factor on protease production, followed by B-starch with moderate influence, while other factors and interactions showed no significant effects. The model's high coefficient of determination (R^2^ = 0.8718) and low coefficient of variation (C.V. = 13.32%) indicate a good fit and moderate precision, respectively. The Pareto chart ranks Meat and bone, Starch, and CaCO_3_ as the top three factors affecting enzyme activity and total amino acids production, suggesting that optimizing these may enhance protease production.

### Box-Behnken design for peptone optimization using *B.subtilis* strain KEMET024

**Fig. 5:**
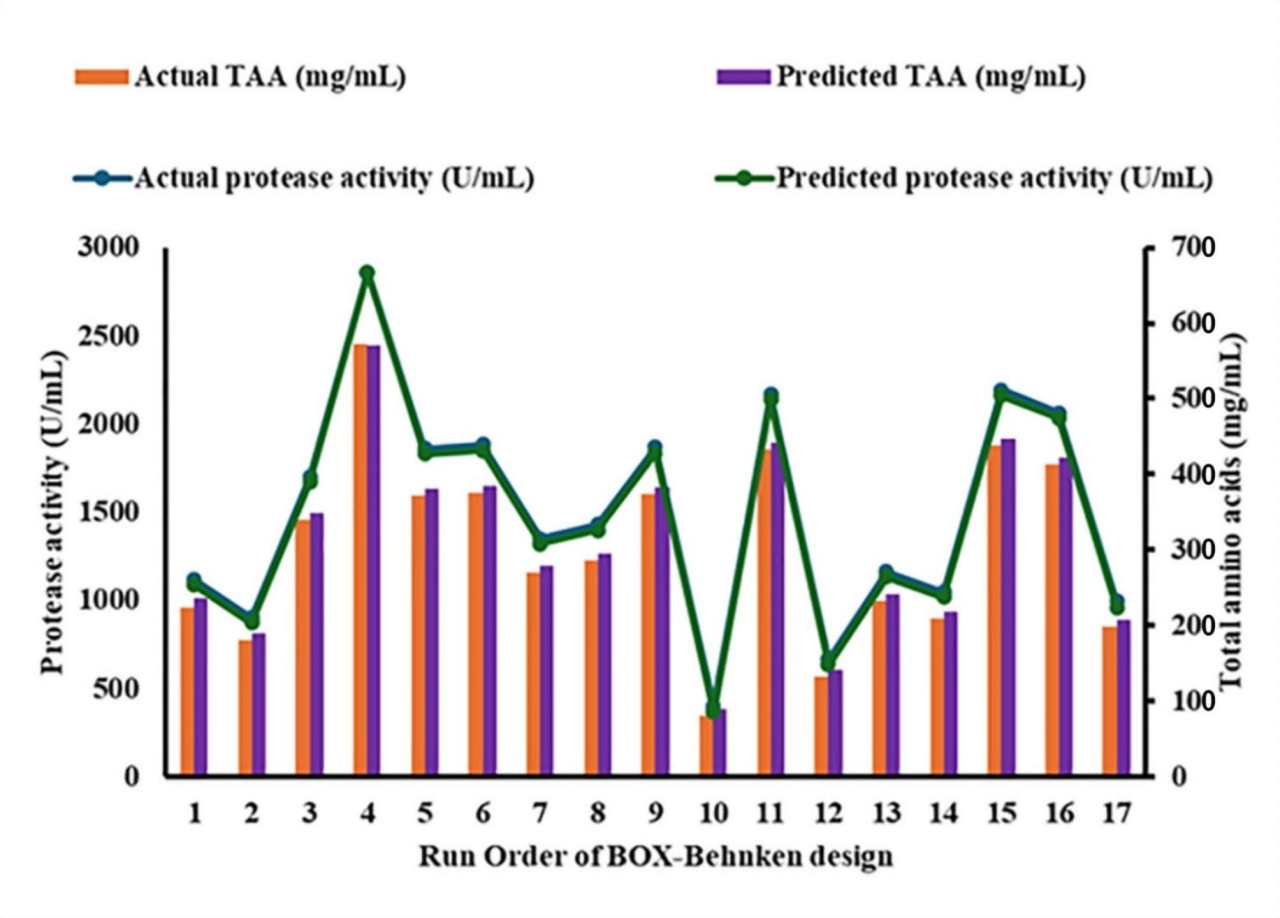
Actual and predicted protease activity and TAA from *B. subtilis* KEMET024 BOX-Behnken experimental runs

**Fig. 6:**
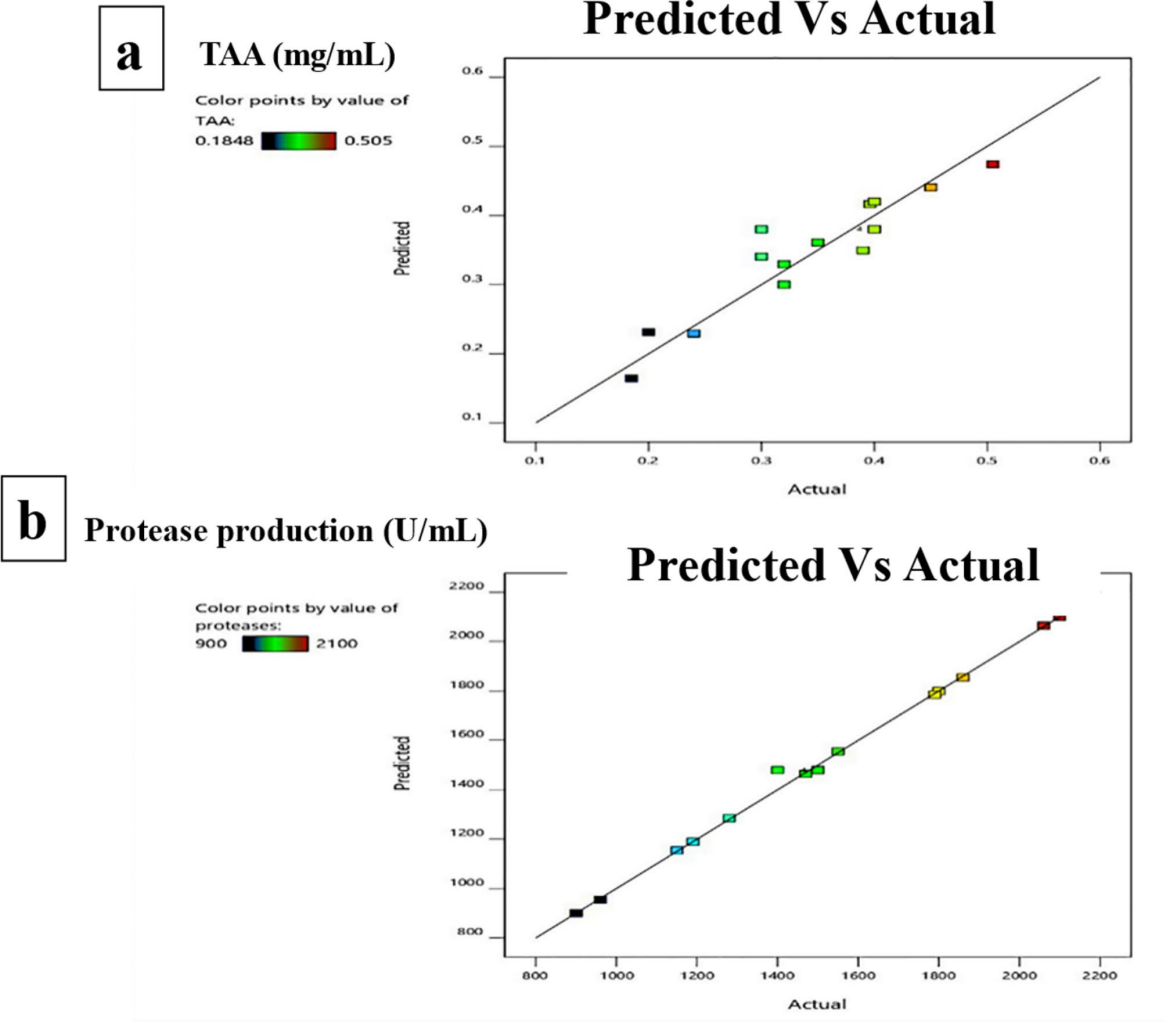
Comparative analysis of predicted and actual TAA concentration and protease activity from *B. subtilis* KEMET024 in a BOX-Behnken Design Experiment

**Fig. 7:**
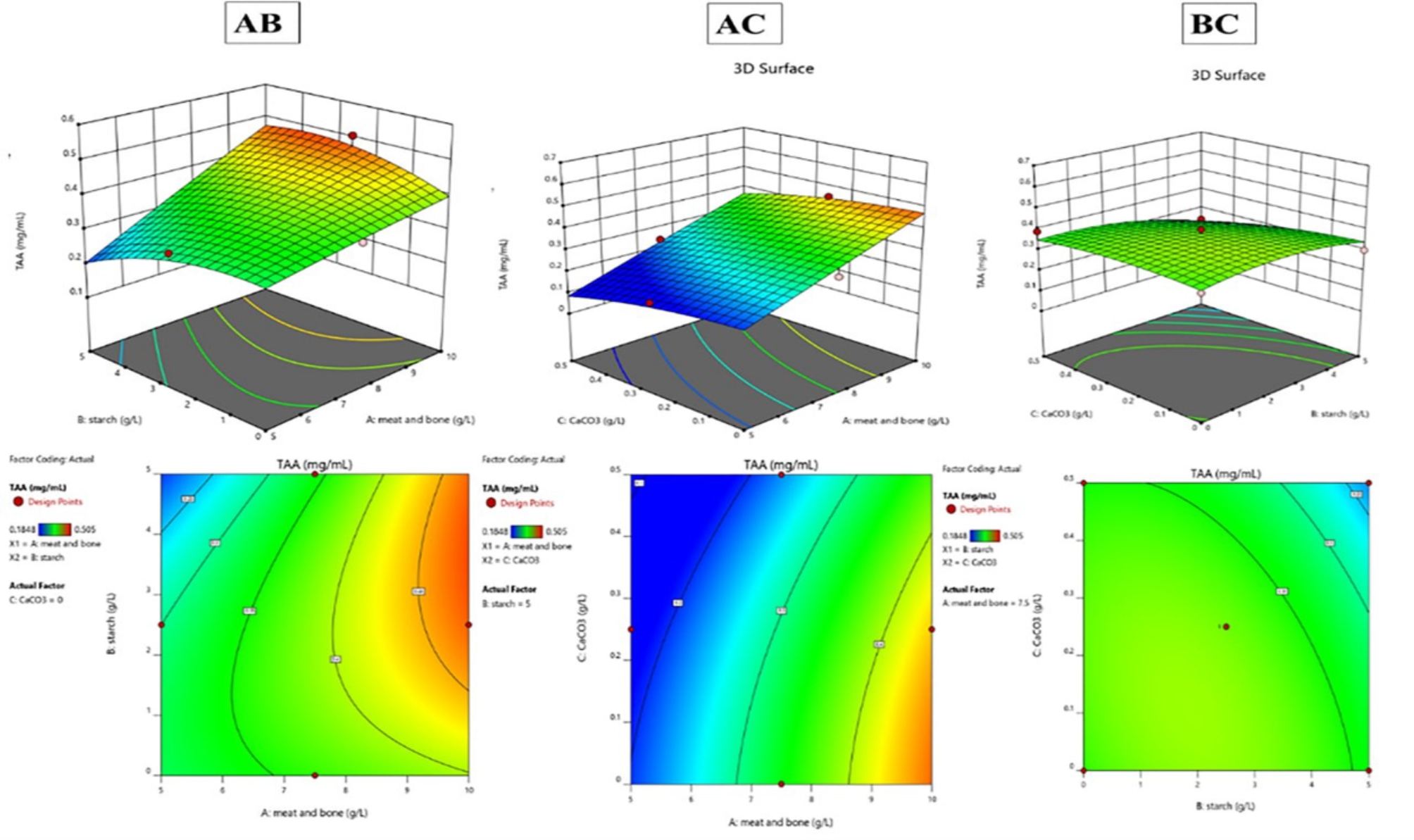
3D surface plots and 2D contour lines for the optimization of TAA from *B. subtilis* KEMET024 using Box-Behnken design of response surface methodology. **A**: meat and bone, **B**: Strach, **C**: CaCO_3_

**Fig. 8:**
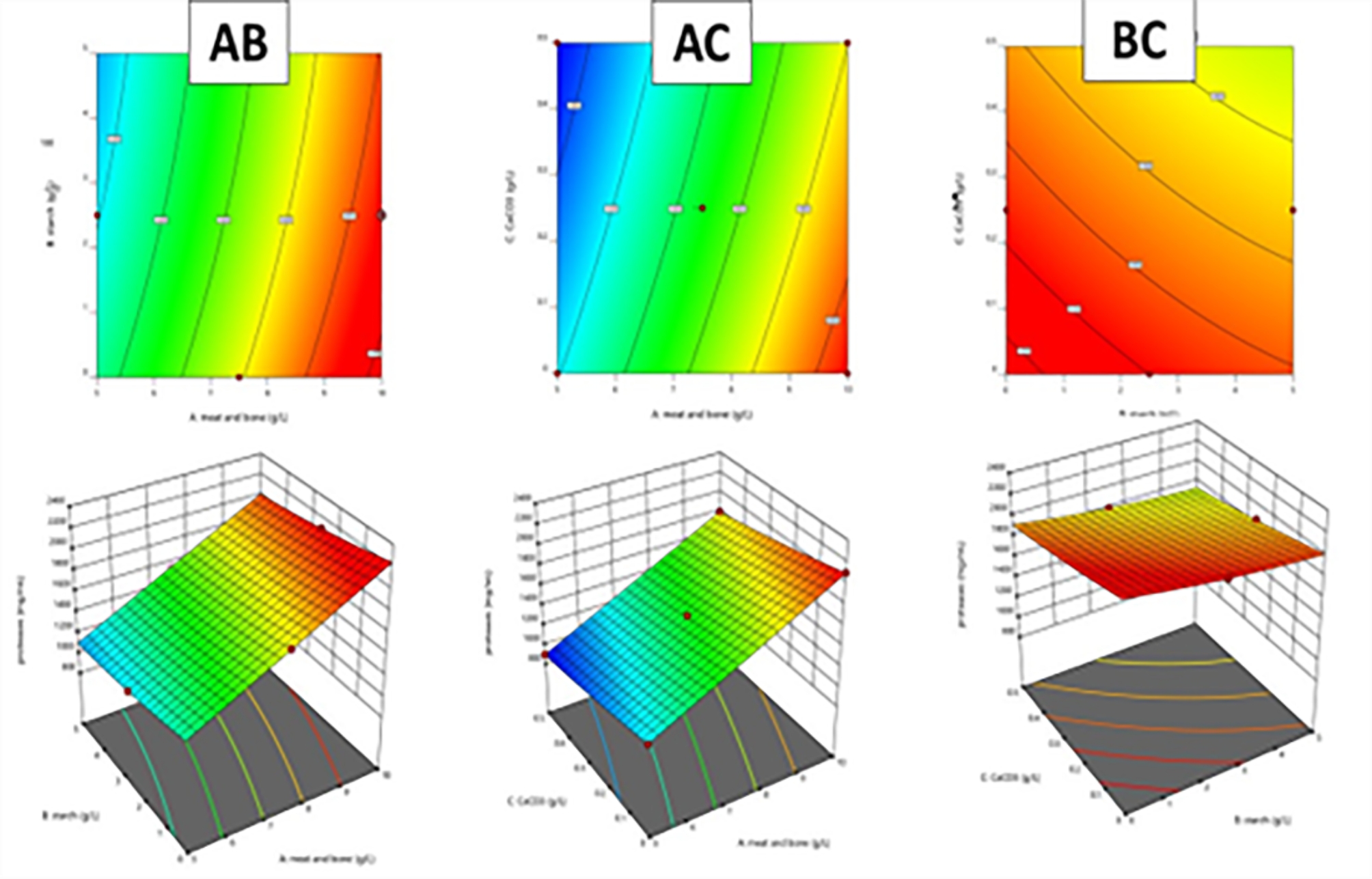
3D surface plots and 2D contour lines for the optimization of protease production from *B. subtilis* KEMET024 using Box-Behnken design of response surface methodology. **A**: meat and bone, **B**: Strach, **C**: CaCO_3_

Figure 5 demonstrates the predictive accuracy of the ANOVA model for total amino acid (TAA) concentration and protease activity, showing a close alignment between actual and predicted values. The model effectively forecasts protease activity that reached 2850 U/mL and 621.5 mg/mL for amino acids production at run 4, while Fig. 6 underscores the importance of continual model adjustment to improve prediction precision. Figures 7 and 8, with 3D surface and contour plots, identify optimal conditions for maximizing TAA and protease, showing that each response may need different conditions. The normal residual plots validate the ANOVA model’s assumptions by confirming that residuals for both TAA and protease follow a normal distribution, supporting the model’s robustness and statistical reliability. The ANOVA analysis of the data reveals significant insights into the factors affecting (TAA) and protease production (Table 2). The Model F-value of 5.29 implies the model is significant. P-values less than 0.0500 indicate model terms are significant. In this case, A (meat and bone) is a significant model term. The model's high coefficient of determination (R^2^ = 0.8718) and low coefficient of variation (C.V. = 13.32%) indicate a good fit and moderate precision, respectively. Coded eqution for total amino acids is Y_TAA_ = 0.38  + 0.0907875 * A  + -0.0351625 * B  + -0.030625 * C  + 0.047275 * AB  + 0.00375 * AC  + -0.025 * BC  + -0.0030125 * A^2  + -0.0392625 * B^2  + -0.0207375 * C^2. Coded equation for Protease production is Y_Protease production_ = 1480  + 452.5 * A  + -102.5 * B  + -147.5 * C  + -2.5 * AB  + -2.5 * AC  + 12.5 * BC  + 1.25 * A^2  + 26.25 * B^2  + 16.25 * C^2.

**Table 2:** Analysis of variance (ANOVA) for the response surface model, evaluating the significance of meat and bone meal, starch, CaCO_3_ concentrations, and their interactions on TAA and protease production

	Source		Sum of Squares		df	Mean Square	F-value	p-value	
TAA production	Model		0.1037		9	0.0115	5.29	0.0195	significant
	A-meat and bone		0.0659		1	0.0659	30.26	0.0009	
	B-starch		0.0099		1	0.0099	4.54	0.0706	
	C-CaCO_3_		0.0075		1	0.0075	3.44	0.1059	
	AB		0.0089		1	0.0089	4.10	0.0825	
	AC		0.0001		1	0.0001	0.0258	0.8769	
	BC		0.0025		1	0.0025	1.15	0.3196	
	A^2^		0.0000		1	0.0000	0.0175	0.8984	
	B^2^		0.0065		1	0.0065	2.98	0.1280	
	C^2^		0.0018		1	0.0018	0.8310	0.3923	
	Residual		0.0153		7	0.0022			
	Lack of Fit		0.0073		3	0.0024	1.21	0.4141	not significant
	Std. Dev.: 0.0467, C.V. %: 13.32, R^2^: 0.8718								
Protease production	Source	Sum of Squares		df	Mean Square	F-value	p-value	< 0.0001	Significant
	Model	1.901E + 06		9	2.112E + 05	180.32		< 0.0001	
	A-meat and bone	1.638E + 06		1	1.638E + 06	1398.34		< 0.0001	
	B-starch	84,050.00		1	84,050.00	71.75		< 0.0001	
	C-CaCO_3_	1.741E + 05		1	1.741E + 05	148.58		0.8880	
	AB	25.00		1	25.00	0.0213		0.8880	
	AC	25.00		1	25.00	0.0213		0.4888	
	BC	625.00		1	625.00	0.5335		0.9424	
	A^2^	6.58		1	6.58	0.0056		0.1595	
	B^2^	2901.32		1	2901.32	2.48		0.3624	
	C^2^	1111.84		1	1111.84	0.9491			
	Residual	8200.00		7	1171.43			0.9906	
	Lack of Fit	200.00		3	66.67	0.0333		< 0.0001	Not significant
	Std. Dev.: 34.23, C.V. %: 2.28, R^2^: 0.9957								

### Chemical analysis of commercial peptone

As shown in Table 3 and Fig. 1S, the table compares commercial and produced peptones in terms of amino acid content. Notably, Aspartic Acid and Glutamic show a significant increase in the produced alternative, with Aspartic Acid having a 37.745% amount compared to 13.31% in commercial peptones, and glutamic showing aremarkable 90.876% over 14.71%. Glycine and Alanine also exhibit higher percentages in the alternative, with Glycine at 117.272% and Alanine at 50.373%, indicating a more than twofold increase. Conversely, Methionine and Histadine are present in lower amounts in the alternative, with 2.066% and 4.563% respectively, compared to 14.92% and 15.52% in commercial peptones. Overall, the produced alternative shows a higher total amino acid content of 621.556 mg/g compared to 230.81 mg in commercial peptones, suggesting a more concentrated source of amino acids.

**Table 3:** Comparative analysis of amino acid profiles in commercial and the produced peptones from *B. subtilis* KEMET024

	Compound Name	Commercial peptones				Meat and bone byproduct peptones		
		Reten. Time (min)	Response	Amount (mg/mL)	Amount%	Reten. Time (min)	Response	Amount (mg/mL)
1	Aspartic Acid	8.04	6378.363	13.31	5.8	7.882	9606.012	37.745
2	Threonine	9.778	4119.598	11.91	5.2	9.647	2071.406	11.276
3	Serine	10.444	4485.019	10.51	4.6	10.331	5242.779	23.134
4	Glutamic	11.698	4967.533	14.71	6.4	11.598	16,297.715	90.876
5	Proline	13.409	251.733	11.51	5	13.289	980.607	84.39
6	Glycine	16.767	4752.982	7.507	3.3	16.653	39,431.38	117.272
7	Alanine	17.991	3795.559	8.909	3.9	17.831	11,397.143	50.373
8	Cystine	18.756	178.427	12.015	5.2	20.269	121.281	15.378
9	Valine	22.647	6245.108	11.72	5.1	22.642	4480.209	15.832
10	Methionine	24.458	5004.703	14.92	6.5	24.411	368.028	2.066
11	Isoleusine	26.291	4967.239	13.12	5.7	26.251	2041.927	10.156
12	Leucine	27.549	4884.543	13.12	5.7	27.489	4642.649	23.482
13	Tyrosine	30.54	4680.903	18.12	7.9	30.464	643.414	4.69
14	Phenylalanine	31.756	4786.148	16.52	7.2	31.671	2380.715	15.473
15	Histadine	35.256	6124.264	15.52	6.7	35.193	956.174	4.563
16	Lysine	39.573	6758.907	14.62	6.3	39.491	5704.291	23.234
17	Ammonium	41.796	16,243.164	5.349	2.3	41.533	38,855.987	24.094
18	Arginine	43.373	5909.358	17.42	7.5	43.271	12,164.46	67.523
		Total		230.81	100			621.556

	Compound Name	Commercial				Produced alternative		
		Reten. Time (min)	Response	Amount (mg/mL)	Amount% (%)	Reten. Time (min)	Response	Amount (mg/mL)
	Proline	13.409	1535.775	2.989	100	13.289	5431.366	10.571
		Total		2.989	100			10.571

### Toxicological properties of produced peptone against normal human skin fibroblast (HSF)

**Fig. 9:**
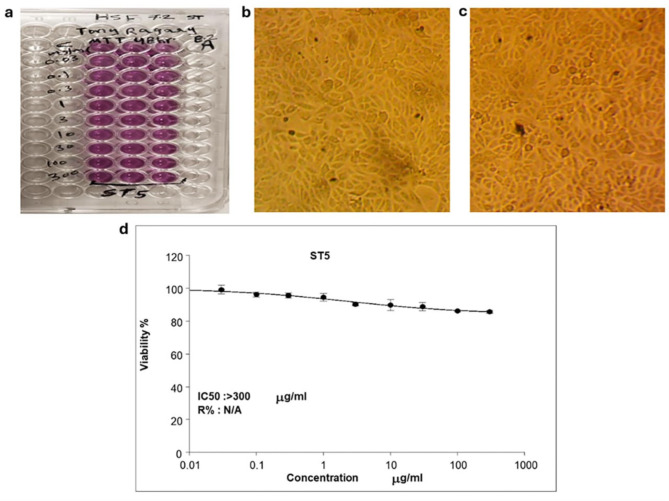
Dose-dependent cell viability and cytotoxicity profile of peptones from *B. subtilis* KEMET024 using MTT assay against normal HSF cell line. **a** cell viability ELISA plate. **b**: control, **c** viability of cells at 300 concentration of produced peptones showed normal aggregated cells

Figure 9 illustrates a cell viability assessment, where at the minimal concentration of 0.03 µg/mL, cells thrive with a high survival rate of 99.09 ± 2.73%, paralleling the blank control’s score of 100 ± 0.00%. As the concentration incrementally ascends to 0.1, 0.3, and 1 µg/mL, a gentle depletion in cell viability is noted, yielding rates of 96.18 ± 1.41%, 95.58 ± 1.55%, and 94.49 ± 2.35% respectively. In contrast, higher concentrations of 3, 10, and 30 µg/mL mark a greater decrease in viability, descending to 90.29 ± 1.13%, 89.75 ± 3.32%, and 88.74 ± 2.60% respectively, indicative of escalated cytotoxic effects. The pattern reaches its zenith at a concentration of 300 µg/mL, where cell viability sharply declines to its lowest at 85.61 ± 0.82%, significantly trailing behind the blank control and lower concentrations, thereby accentuating the increased cytotoxicity at this elevated level.

### Characterization of the proteolytic activity of the produced peptones

**Fig. 10:**
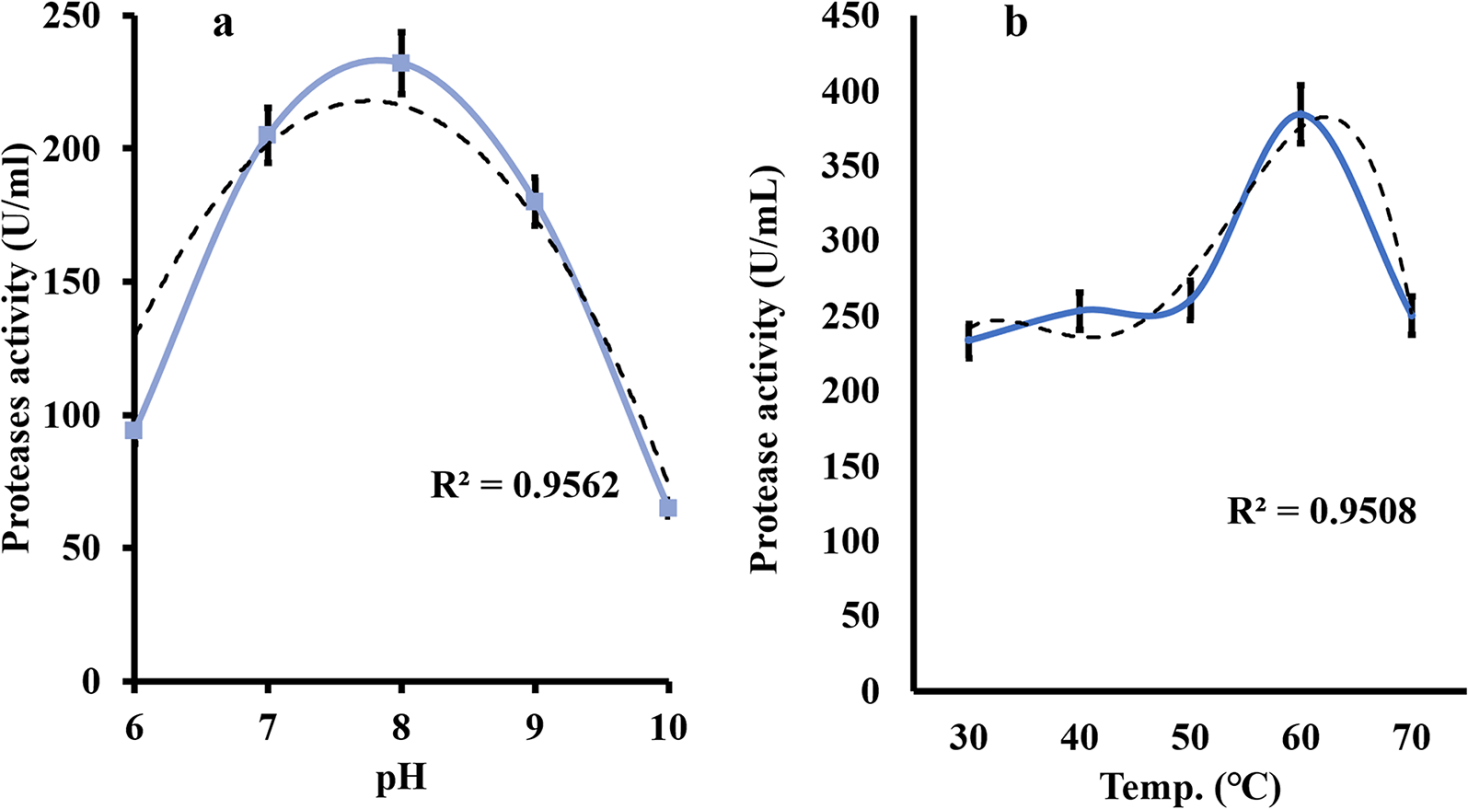
Protease activity characterization under optimal pH and temperature conditions of *B. subtilis* KEMET024

As shown in Fig. 10 The results from the study on protease activity derived from feather degradation reveal that the enzyme operates optimally at a pH of around 8 and a temperature range of 50–60°C, as evidenced by the peak activities of 250 U/mL and the bell-shaped curves on the graphs. The high R^2^ values of 0.95 for pH and 0.87 for temperature indicate a strong correlation between these factors and enzyme activity.

### Effect of poultry waste substrate concentration on enzyme velocity

**Fig. 11:**
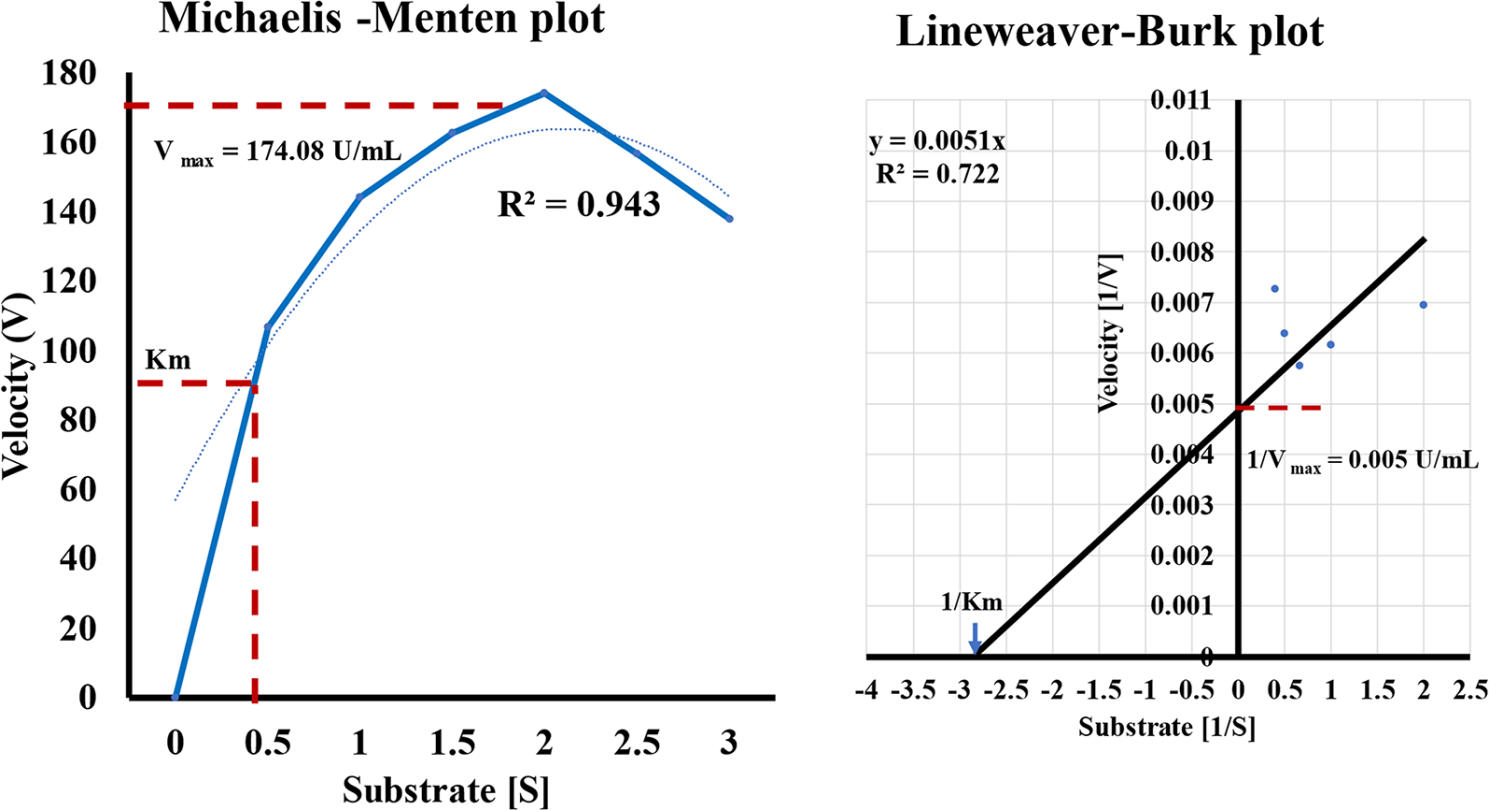
Michalis-Menten and Lineweaver–Burk plots for protease activity and peptone production using *B. subtilis* KEMET024

As shown in Fig. 11, the velocity of the enzymatic reaction (first-order) increased with rising substrate concentration until saturation of the active sites occurred. Protease activity gradually enhanced with increasing poultry waste substrate up to 3 g/L, giving a maximum value of 174.08 U/mL at higher substrate levels, competitive binding reduced the reaction rate as sites became saturated. The kinetic parameters calculated from the Michaelis–Menten and Lineweaver–Burk plots were a Km of 0.5 mg/mL and a Vmax of 174.08 U/mL.

### Application of produced peptone from *B. subtilis* strain KEMET024 as the sole source of nitrogen

**Fig. 12:**
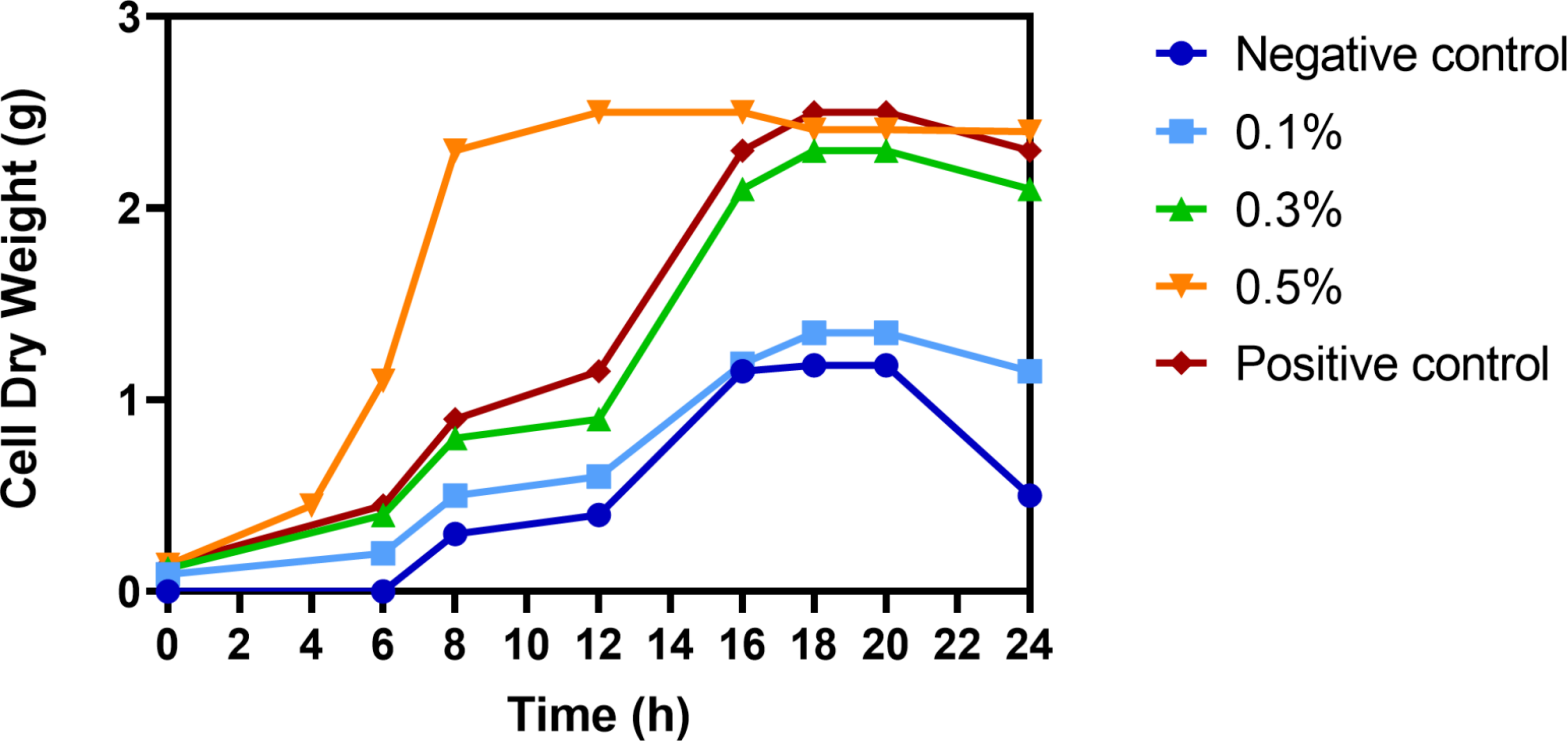
Growth curve of *B. subtilis* ATCC 6051 grown on minimal medium broth using different concentration of produced peptone incubated at 30 °C for 24h at 150 rpm

The growth curve depicted in Fig. 12 demonstrates the effectiveness of peptone produced from *B. subtilis* strain KEMET024 as a nitrogen source, with varying concentrations (0.1%, 0.3%, and 0.5%) tested over 24 h at 30°C. The 0.5% peptone concentration showed superior performance, reaching the highest cell dry weight of approximately 2.5g and exhibiting rapid growth between 4–8 h, even outperforming the positive control. The 0.3% concentration performed similarly to the positive control, while 0.1% showed minimal growth comparable to the negative control. All conditions followed typical bacterial growth phases, with most reaching stationary phase around 18–20 h, followed by a slight decline. These results strongly indicate that the produced peptone, particularly at 0.5% concentration, is an effective nitrogen source for *B. subtilis* cultivation, demonstrating its potential for biotechnological applications.

## Discussion

The study successfully isolated several proteolytic bacterial strains from poultry waste, where isolates P3, P4, P5, and P6 exhibited significantly higher protease activity on both skim milk agar and feather agar compared to the control strain *B. subtilis* ATCC 6051. This finding aligns with previous studies that have identified poultry waste as a rich source of proteolytic microorganisms, particularly *Bacillus* species, due to their ability to degrade complex proteinaceous substrates (Nassar 2015a).

The 16S rRNA gene sequencing and subsequent phylogenetic analysis revealed that the highly potent isolate P6 shared a 94% similarity with the *B. subtilis* strain KEME T024 (GenBank accession number PP694485.1). This identification corroborates numerous reports highlighting *B. subtilis* as a prolific producer of various extracellular enzymes, including proteases. (Nassar 2015a). The distinct separation of the isolate from marine *Bacillus* strains suggests its terrestrial origin and potential adaptation to the poultry waste environment.

The Plackett–Burman design and subsequent Box-Behnken response surface methodology effectively optimized the peptone production process by identifying critical factors and their interactions. (Abd-Elhalim 2023a; Nassar 2015b). The study revealed that the concentration of meat and bone meal had the most significant impact on both total amino acid (TAA) and protease production. (Abu-Hussien and Mohamed 2020). This finding aligns with previous studies that have emphasized the importance of protein-rich substrates, such as meat and bone meal, in enhancing protease and peptone yields. (Wang 2016).

The high correlation between predicted and actual values for TAA and protease activity, along with the normal distribution of residuals, validates the robustness of the ANOVA model employed. These statistical tools have been widely used in optimizing bioprocesses, including enzyme production and fermentation processes. (Abd-Elhalim 2023a).

The produced peptone exhibited a higher TAA content (621.556 mg/g) compared to commercial peptones (230.81 mg/g), with notable increases in aspartic acid, glutamic acid, glycine, and alanine concentrations. This improved amino acid profile can be attributed to the efficient hydrolysis of the poultry waste substrate by the proteolytic bacteria. Similar observations have been reported in studies utilizing waste materials, such as fish by-products and agricultural residues, for peptone production (Wang 2016).

The dose-dependent cell viability assessment using the MTT assay against normal human skin fibroblast (HSF) cells revealed minimal cytotoxicity at lower concentrations (≤ 1 mg/mL) of the produced peptone, with cell viability exceeding 94%. However, higher concentrations (≥ 3 mg/mL) exhibited increased cytotoxic effects, with cell viability dropping to approximately 85% at the highest concentration tested (300 mg/mL). This finding emphasizes the need for careful evaluation of the produced peptone's cytotoxicity and the establishment of safe concentration ranges for potential applications. Similar observations have been reported in studies evaluating the cytotoxicity of peptones derived from various sources. (Veerapandian 2020).

The study characterized the proteolytic activity of the produced protease from *B. subtilis*, revealing optimal pH and temperature conditions of around pH 8 and 50–60°C, respectively. These findings align with reports on keratinolytic proteases from *Bacillus* species, which often exhibit alkaline pH and thermophilic temperature optima (Nassar 2015a). The Michaelis–Menten and Lineweaver–Burk plots provided insights into the enzyme's kinetics and substrate binding behavior, further supporting the potential industrial applications of such enzymes in waste degradation and by-product recovery. In conclusion, study successfully isolated and characterized a novel *B. subtilis* strain KEMET024 with remarkable proteolytic capabilities, particularly in degrading both milk protein, meat and bone poultry waste, and feather substrates. The strain demonstrated superior protease activity compared to the control *B. subtilis* ATCC 6051, especially on feather agar. Through statistical optimization using Plackett–Burman and Box-Behnken designs, meat and bone meal emerged as the most significant factor affecting both protease production and total amino acid yield. The optimized conditions led to the production of peptones with notably higher amino acid content (621.556 mg/g) compared to commercial peptones (230.81 mg/g), with particularly elevated levels of glutamic acid, glycine, and aspartic acid. Toxicological assessment against HSF revealed minimal cytotoxicity even at high concentrations, with cell viability remaining above 85% at 300 µg/mL. The characterized protease showed optimal activity at pH 8 and 50–60°C, making it suitable for industrial applications. These findings demonstrate the potential of *B. subtilis* KEMET024 for sustainable bioconversion of poultry waste into valuable peptone products, offering a cost-effective and environmentally friendly alternative to commercial peptones while maintaining safety for biological applications.

## Supplementary Information

Additional file1 (PDF 146 kb)

## Data Availability

The raw data and analyzed data used during the current study are available from the corresponding author upon reasonable request. *B.subtilis* ATCC 6051 was provided by the Microbial Resources Center, Faculty of Agriculture, Ain Shams University, Cairo, Egypt, and was deposited in the following strain provider. *B.subtilis* ATCC 6051 (https://www.atcc.org/products/6051) *Bacillus subtilis* KEMET024 was deposited in GenBank with gene accession number PP694485.1 and deposited in MIRCEN culture collection, Cairo, Egypt as *Bacillus subtilis* EMCC 998871.
