# Supplementary material for: Optimization and characterization studies of poultry waste valorization for peptone production using a newly Egyptian Bacillus subtilis strain
Source: AMB Express. 2025 Jan 13;15:9. doi: 10.1186/s13568-024-01794-1 (PMC11730028; doi:10.1186/s13568-024-01794-1)
Supplement: Supplementary file 1 — Additional file1 (PDF 146 kb) [file 13568_2024_1794_MOESM1_ESM.pdf]

Journal name: **AMB express**

Optimization and characterization studies of Poultry Waste Valorization for Peptone Production Using a newly Egyptian *Bacillus subtilis* KEMET024 by Box-Behnken Design

Hajar Saeed[1], Anthony Ragaey[1], Ziad Samy[1], Viola Ashraf[1], Aly ElMostafa[1], Norhan Ahmad[1], Enjy Bebawy[1], Nour ElHoda M. Sorour[2], Salwa M. El-Sayed[3], Ashraf Bakry[2], Naglaa Ebeed[2], Hesham El-hariry[4], Thanaa El-Noby[5], Samah H. Abu-Hussien[6]*

[1]Biotechnology Program, New Programs Administration, Faculty of Agriculture, Ain Shams University, P.O. Box 68-Hadayek Shoubra, Cairo, 11241, Egypt

[2]Department of Genetics, Faculty of Agriculture, Ain Shams University, P.O. Box 68-Hadayek Shoubra, Cairo, 11241, Egypt

[3]Department of Biochemistry, Faculty of Agriculture, Ain Shams University, P.O. Box 68-Hadayek Shoubra, Cairo, 11241, Egypt

[4]Department of Food science, Faculty of Agriculture, Ain Shams University, P.O. Box 68-Hadayek Shoubra, Cairo, 11241, Egypt

[5]Department of Agricultural Economics, Faculty of Agriculture, Ain Shams University, P.O. Box 68-Hadayek Shoubra, Cairo, 11241, Egypt

[6]Department of Agricultural Microbiology, Faculty of Agriculture, Ain Shams University, P.O. Box 68-Hadayek Shoubra, Cairo, 11241, Egypt

Correspondence: samah_hashem1@agr.asu.edu.eg

Table S1: Nutritional and environmental factors with their low and high levels for the screening of peptone production using P6 isolate by Plackett-Burman design.

Factor	Name	Units	Minimum	Maximum
A	Feather meal	g/L	10.00	15.00
B	meat and bone	g/L	10.00	15.00
C	Starch	g/L	0.0000	20.00
D	Casein	g/L	0.0000	15.00
E	$CaCO_3$	g/L	0.2000	0.5000
F	KH_2PO_4	g/L	0.2000	0.5000
G	pH		6.00	8.00
H	temp.	°C	25.00	40.00

J	Inoculum size	%	3.00	7.00
K	Agitation	rpm	100.00	200.00
L	incubation time	days	24.00	48.00

I is a dummy factor by default using Design Expert 12 software.

Table S2: Plackett-Burman design matrix for peptone optimization by *B.subtilis* strain KEMET024

	Factor A: Feather meal (g/L)	Factor B: Meat and bone (g/L)	Factor C: Starch (g/L)	Factor D: Casein (g/L)	Factor E: $CaCO_3$ (g/L)	Factor F :KH_2PO_4 (g/L)	Factor G: pH	Factor H: Temp (°C)	Factor J: Inoculum size %	Factor K: Agitation rpm	Factor L: incubation days
1	+1	+1	-1	+1	+1	+1	-1	-1	-1	+1	-1
2	-1	-1	+1	-1	+1	+1	-1	+1	+1	+1	-1
3	-1	-1	-1	+1	-1	+1	+1	-1	+1	+1	+1
4	-1	-1	-1	-1	-1	-1	-1	-1	-1	-1	-1
5	+1	+1	+1	-1	-1	-1	+1	-1	+1	+1	-1
6	0	0	0	0	0	0	0	0	0	0	0
7	0	0	0	0	0	0	0	0	0	0	0
8	0	0	0	0	0	0	0	0	0	0	0
9	0	0	0	0	0	0	0	0	0	0	0
10	0	0	0	0	0	0	0	0	0	0	0
11	0	0	0	0	0	0	0	0	0	0	0
12	+1	+1	-1	-1	-1	+1	-1	+1	+1	-1	+1
13	+1	-1	+1	+1	-1	+1	+1	+1	-1	-1	-1
14	-1	+1	+1	+1	-1	-1	-1	+1	-1	+1	+1
15	+1	-1	-1	-1	+1	-1	+1	+1	-1	+1	+1
16	+1	-1	+1	+1	+1	-1	-1	-1	+1	-1	+1
17	-1	+1	+1	-1	+1	+1	+1	-1	-1	-1	+1
18	-1	+1	-1	+1	+1	-1	+1	+1	+1	-1	-1

Table S3: Nutritional factors with their low and high levels and their statistical screening matrix for the optimization of peptone production using *B.subtilis* strain KEMET024 by Box-Behnken design.

**Nutritional factors with their low and high levels**

Factor	**Name**	**Level**	**Low Level**	**High Level**
A	Meat and bone	10.00	5.00	10.00
B	starch	0.0000	0.0000	5.00
C	$CaCO_3$	0.2500	0.0000	0.5000
**Statistical screening of nutritional factors using Box-Behnken (PBD) design**				
Run	**Meat and bone**	**Starch**	**$CaCO_3$**	
1	0	0	0	
2	-1	0	+1	
3	0	0	0	
4	0	+1	-1	
5	0	0	0	
6	0	0	0	
7	0	+1	+1	
8	0	-1	-1	
9	0	-1	+1	
10	0	0	0	
11	+1	-1	0	
12	-1	+1	0	
13	-1	-1	0	
14	-1	0	-1	
15	+1	0	+1	
16	+1	0	-1	
17	+1	+1	0	

Figure S1: Comparative analysis of amino acid Profiles in commercial peptones and produced alternative from *B.subtilis* strain KEMET024

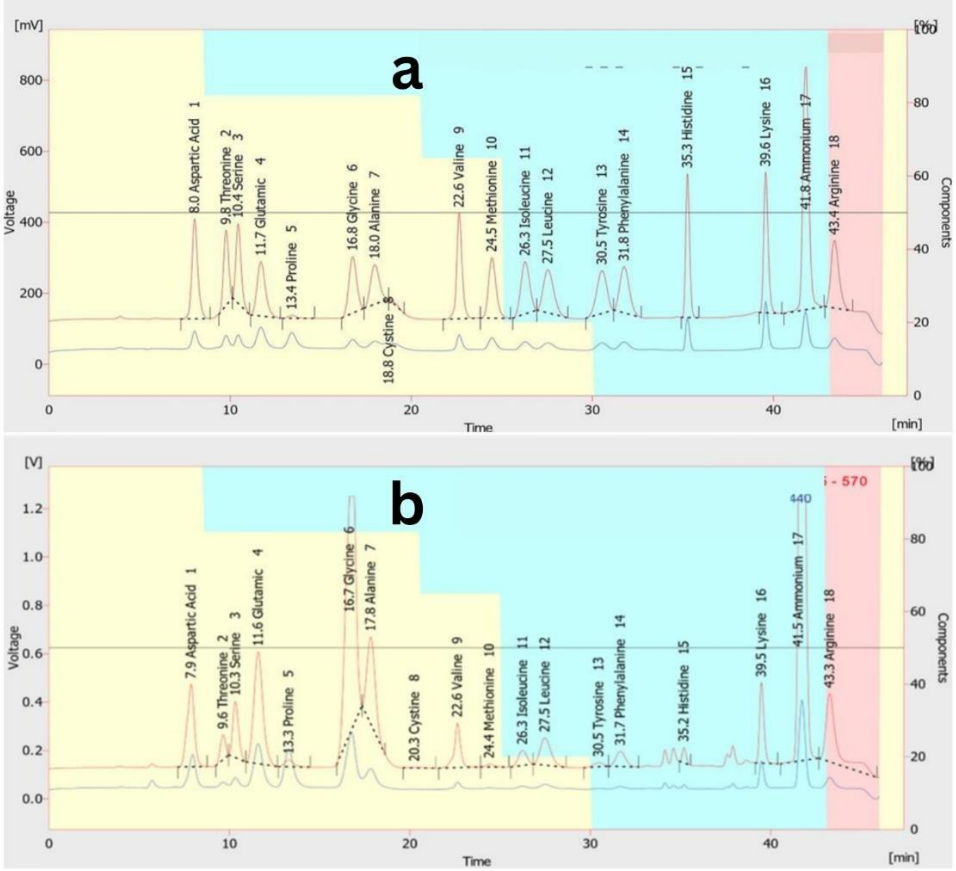
a
b
8.0 Aspartic Acid 1
9.8 Threonine 2
10.4 Serine 3
11.7 Glutamic 4
13.4 Proline 5
16.8 Glycine 6
18.0 Alanine 7
18.8 Cystine 8
22.6 Valine 9
24.5 Methionine 10
26.3 Isoleucine 11
27.5 Leucine 12
30.5 Tyrosine 13
31.8 Phenylalanine 14
35.3 Histidine 15
39.6 Lysine 16
41.8 Ammonium 17
43.4 Arginine 18
7.9 Aspartic Acid 1
9.6 Threonine 2
10.3 Serine 3
11.6 Glutamic 4
13.3 Proline 5
16.7 Glycine 6
17.8 Alanine 7
20.3 Cystine 8
22.6 Valine 9
24.4 Methionine 10
26.3 Isoleucine 11
27.5 Leucine 12
30.5 Tyrosine 13
31.7 Phenylalanine 14
35.2 Histidine 15
39.5 Lysine 16
41.5 Ammonium 17
43.3 Arginine 18
[mV]
[V]
Voltage
Time
[min]
[%]
Components
440
